# High-Dose Accelerated Bilateral Theta Burst Stimulation for Depression and Anxiety: The Seville Protocol

**DOI:** 10.1192/j.eurpsy.2025.2127

**Published:** 2025-08-26

**Authors:** M. Martín-Bejarano, Á. Moleón, P. Álvarez de Toledo, I. Pérez-Aquino, J. Narbona-Antúnez, J. Torres-Pereira, M. García-Ferriol

**Affiliations:** 1 Institulo Andaluz de Salud Cerebral; 2 Hospital Virgen del Rocío, Sevilla, Spain

## Abstract

**Introduction:**

Treatment resistance affects 20-60% of patients, leading to substantial personal and economic impact. Repetitive transcranial magnetic stimulation (rTMS) is effective, with theta burst stimulation (TBS) providing similar benefits more efficiently.

**Objectives:**

To assess high-dose TBS effectiveness and to explore how demographic and clinical factors influence treatment outcomes.

**Methods:**

Accelerated high-dose (30 sessions) cTBS and iTBS was administered targeting the right and left dorsolateral prefrontal cortex (DLPFC) respectively (3600 pulses per session), with MRI-guided neuronavigation. Pre- and post-treatment HAM-D and HAM-A scores were analyzed with mixed-effects models. Response and remission rates were further examined using generalized linear models (GLM). All analyses were conducted using the R Studio.

**Results:**

The study included a total of 101 participants, of whom 89 had data available for HAM-D (56 [38.8–65] years; 69.7% females), and 82 had data available for HAM-A (56 [39–65] years; 70.7% females). 29.2% achieved HAM-D remission, 22% achieved HAM-A remission, with response rates of 46.1% for depression and 50% for anxiety.

Mixed-effects models showed a highly significant reduction in both HAM-D and HAM-A scores after TMS treatment (HAM-D: β = -12, p = 2.2e−15; HAM-A: β = -14.484, p = 1.1×10−14) (Fig. 1). For HAM-D, family history was associated with reduced treatment effectiveness (β = 5.302, p = 0.011). Sex also influenced HAM-D scores, with males showing a greater response than females (p = 0.018), although this trend was only marginally significant for HAM-A (p = 0.073).

**Fig. 1.** Pre- and post-treatment scores on the HAM-D and HAM-A showing significant reductions following rTMS.

The GLM analysis for HAM-D and HAM-A remission did not reveal statistically significant overall results. However, specific predictors were significantly associated with treatment response. A family history of mental health conditions was linked to a lower likelihood of response, based on HAM-D (OR = 0.058, p = 0.016) and HAM-A (OR = 0.074, p = 0.049). Age was a significant predictor for response on both HAM-D (OR = 1.1, p = 0.048) and HAM-A (OR = 1.115, p = 0.032) (Fig. 2). Additionally, regarding employment status individuals identified as “Housekeeper” or “Retired” had reduced likelihood of positive response (p < 0.05).

**
Figure 2.** Influence of age on HAMA and HAM-D response outcomes in patients undergoing TMS treatment.

**Image:**

**Figure:**
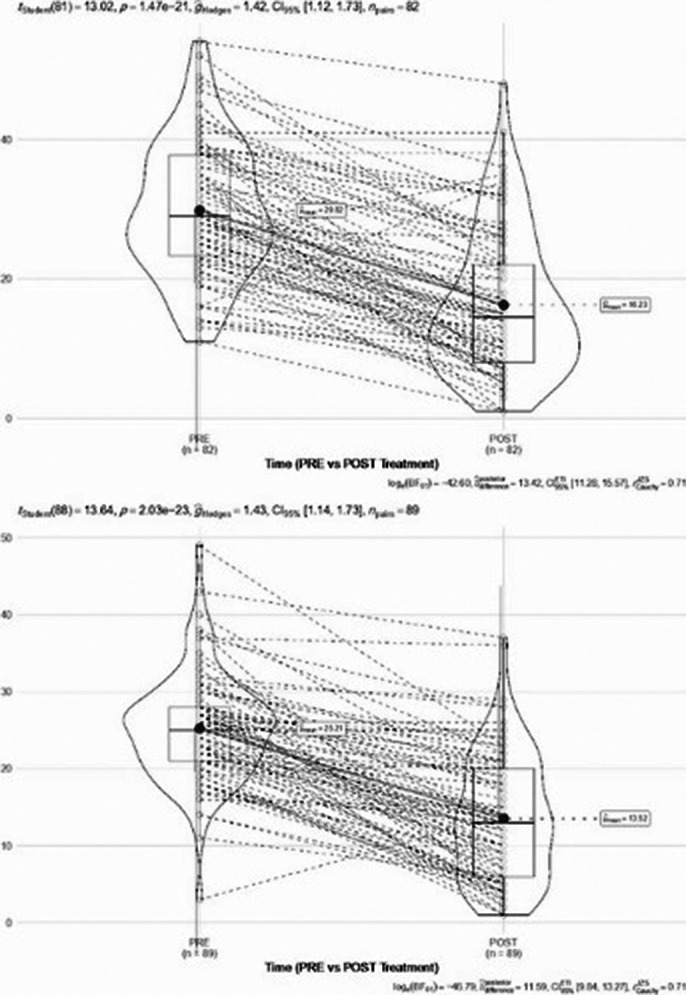

**Image 2:**

**Figure:**
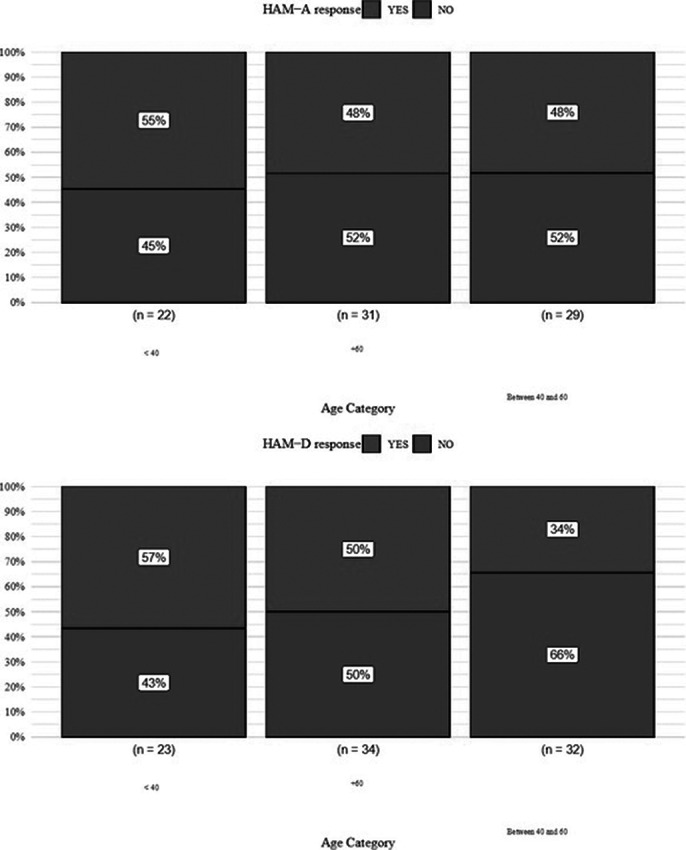

**Conclusions:**

High-dose accelerated bilateral TBS using the Sevilla Protocol significantly reduced depression and anxiety symptoms in treatment-resistant patients, with notable response and remission rates. Family history, age, and certain employment statuses significantly influenced treatment response, suggesting that TBS may benefit from tailored approaches. Larger, balanced samples are needed to confirm these findings and improve prediction models.

**Disclosure of Interest:**

None Declared

